# Genomic landscape of 891 *RET* fusions detected across diverse solid tumor types

**DOI:** 10.1038/s41698-023-00347-2

**Published:** 2023-01-23

**Authors:** Vamsi Parimi, Khaled Tolba, Natalie Danziger, Zheng Kuang, Daokun Sun, Douglas I. Lin, Matthew C. Hiemenz, Alexa B. Schrock, Jeffrey S. Ross, Geoffrey R. Oxnard, Richard S. P. Huang

**Affiliations:** 1 Foundation Medicine, Inc, Cambridge, MA USA; 2 Department of Pathology and Urology, State University of New York (SUNY) Upstate Medical University, Syracuse, New York, NY USA

**Keywords:** Predictive markers, Tumour biomarkers

## Abstract

In this study, we report the clinicopathologic and genomic profiles of 891 patients with *RET* fusion driven advanced solid tumors. All patient samples were tested using a tissue-based DNA hybrid capture next generation sequencing (NGS) assay and a subset of the samples were liquid biopsies tested using a liquid-based hybrid capture NGS assay. *RET* fusions were found in 523 patients with NSCLC and in 368 patients with other solid tumors. The two tumor types with the highest number of *RET* fusion were lung adenocarcinoma and thyroid papillary carcinoma, and they had a prevalence rate 1.14% (455/39,922) and 9.09% (109/1199), respectively. A total of 61 novel fusions were discovered in this pan-tumor cohort. The concordance of *RET* fusion detection across tumor types among tissue and liquid-based NGS was 100% (8/8) in patients with greater than 1% composite tumor fraction (cTF). Herein, we present the clinicopathologic and genomic landscape of a large cohort of *RET* fusion positive tumors and we observed that liquid biopsy-based NGS is highly sensitive for *RET* fusions at cTF ≥1%.

## Introduction

Rearranged during transfection *(RET)*, located near the centromere on the long arm of chromosome 10 (10q11.21), is a proto-oncogene that encodes for a single-pass transmembrane glycoprotein receptor tyrosine kinase (RTK)^1^. *RET* plays a vital role in the embryonic development of the human enteric nervous system and genitourinary tract and is essential for the normal development of cells^2–4^. Somatic *RET* gene alterations, including short variants and fusions, act as pathogenic driver alterations in approximately 2% of solid tumors. *RET* fusions occur in approximately 1% of non-small cell lung cancer (NSCLC) cases and are generally mutually exclusive to other primary driver variants and rearrangements. *RET* fusion-positive lung adenocarcinomas are associated with poor differentiation, solid sub-type, and smaller T stage (≤3 cm) with N2 disease^5^. *RET* fusion-positive NSCLC represents a rare, but clinically actionable, driver alteration class of tumor^6,7^. In addition, *RET* alterations play an essential role in thyroid cancer initiation and progression. *RET* fusions occur in approximately 10% of papillary thyroid carcinomas. While *RET* short variant mutations are pathognomonic of 98% hereditary and 50% of sporadic medullary thyroid cancers (MTC), they are rarely reported in other tumor types.

Early attempts at RET-targeted precision therapy relied on multi-kinase inhibitors such as cabozantinib, vandetanib, and lenvatinib^8–12^. A combination of modest clinical activities with ORR, mPFS, and mOS ranging from 16%–47%, 4.5–7.3 months, and 9.9–11.6 months, and significant toxicity from off-target activities dampened enthusiasm for further development. More recently a new class of RET-selective inhibitors (selpercatinib and pralsetinib) that potently inhibit both wild-type and *RET*-activated (both point mutations and fusions) tumors^13^. Selpercatinib received its accelerated approval by the FDA in May 2020 for metastatic *RET*-fusion+ NSCLC and papillary thyroid cancers and *RET*-mutant medullary thyroid cancer, based on the LIBRETTO-001 trial evaluating its activity in *RET* + advanced solid tumors^14–16^. Similarly, pralsetinib accelerated approval for *RET* fusion-positive NSCLC came in September 2020 and in December 2020 for thyroid cancer based on the ARROW trial^17–19^. Furthermore, in August 2022, Subbiah et al., published their study examining the pan-cancer efficacy of pralsetnib in patients with *RET* fusions from the phase 1/2 ARROW trial^20^. Here, they observed an overall response rate of 57% (study cohort of 29 patients across 12 tumor types) and concluded that responses were observed regardless of tumor types in their study cohort. Most recently (October 2022), in a tumor-agnostic population (*n* = 41, LIBRETTO-001 trial), meaningful clinical activity (objective response rate was 43.9%) in the *RET* fusion positive cohort was shown^21^.

Currently there is a limited understanding in the genomic landscape of NSCLC and other solid tumors harboring *RET* fusions. Numerous prior studies on *RET* gene alterations are constrained by a small sample size where observed distribution frequencies have not reached statistical significance^22–29^. Here, we report the comprehensive molecular portfolio of *RET*-altered cancers among 523 patients with NSCLC and 368 patients with other solid tumors (excluding NSCLC). Our study describes the clinicopathologic and genomic features of *RET* fusion-positive and negative cohorts among NSCLC and other solid tumors.

## Results

### Clinicopathologic characteristics

**Fig. 1:**
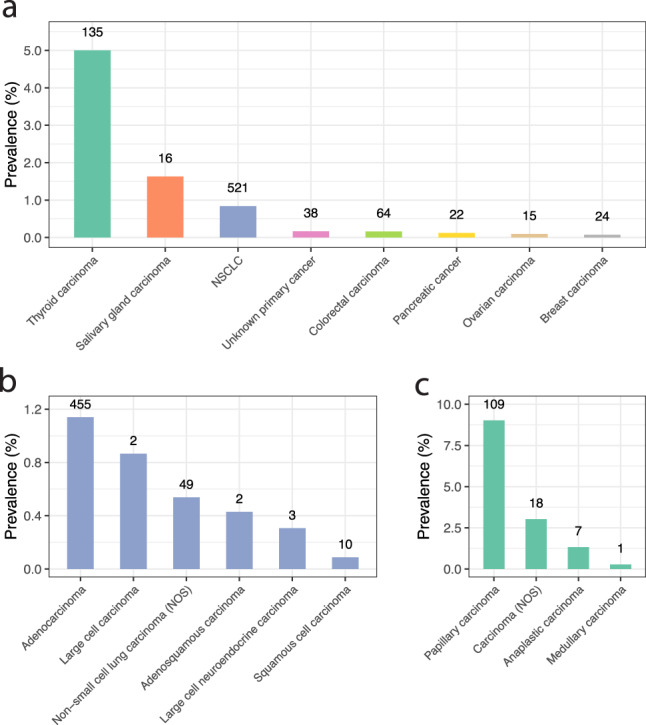
Prevalence of *RET* fusions varied depending on tumor types. **a** The 8 tumor categories had varying prevalence of *RET* fusion (only tumor categories with more than 10 instances were included in figure and tumor categories were composed of tumor types with at least 1 instance as grouped in Supplementary Table 2). The prevalence of *RET* fusions also varied in subtypes of **b** NSCLC and **c** thyroid carcinoma. The two tumor types with the highest number of *RET* fusion were lung adenocarcinoma and thyroid papillary carcinoma and they had a prevalence rate 1.14% and 9.09%, respectively. NOS not otherwise specified. Number on top of bar indicates total number of *RET* fusion cases.

The clinicopathologic and genomic characteristics of 891 patients with *RET* fusions in different cancer types is summarized in Table 1 (523 patients with NSCLC and 368 with other solid tumors. The prevalence of *RET* fusions varied based on tumor type (Fig. 1). The two tumor types with the highest number of *RET* fusion were lung adenocarcinoma and thyroid papillary carcinoma and they had prevalence rates of 1.14% (455/39922) and 9.09% (109/1199), respectively. The other solid tumors cohort consisted of mainly thyroid carcinomas (36.6%, 135/368) and colorectal carcinomas (17.3%, 64/368) followed by carcinomas of unknown primary (10.3%, 38/368), breast carcinomas (6.5%, 24/368), pancreatic carcinomas (5.9%, 22/368) and a wide range of other tumor types (Supplementary Tables 1–2). Among the several histologic subtypes, thyroid papillary carcinoma, colon adenocarcinoma, breast and pancreatic ductal carcinoma, and intra-hepatic cholangiocarcinoma are the most frequent cancers with relatively high prevalence of *RET* fusion positivity. Salivary gland carcinoma represents a tumor type also with a relatively high prevalence (1.6%, 16/982) but with low total number of overall cases, and of these: 4 cases (1.2%, 4/322) were salivary gland adenocarcinoma, 8 cases were (1.8%, 8/446) salivary gland carcinoma (NOS), 3 cases (1.5%, 3/205) were salivary gland duct carcinoma, and the last case (11.1%, 1/9) was a salivary gland mammary analogue secretory carcinoma which harbored a *ETV6-RET* fusion.

**Table 1:** Comparison of clinicopathologic and genomic biomarker characteristics of *RET* fusion-positive NSCLC and *RET* fusion-positive in other solid tumors (excluding NSCLC).

	RET fus + NSCLC	RET fus + other solid tumors	*p* value
	(*n* = 523)	(*n* = 368)	
Age median years [IQR]^a^	64 [55–71]	61 (42–69)	<0.001
Sex, female/male (%)	293/230 (56%/44%)	211/157 (57%/43%)	0.731
Specimen site			
Primary	249 (48%)	163 (44%)	0.623
Metastatic	274 (52%)	155 (42%)	0.140
Cancers of unknown primary	0	38 (10%)	n/a
Unknown	0 (0%)	12 (3.3)	<0.001
Genetic ancestry^b^			
African	41 (7.8%)	35 (9.5%)	1
Central and South American	45 (8.6%)	58 (16%)	0.007
East Asian	55 (11%)	14 (3.8%)	0.001
European	372 (71%)	254 (69%)	1
South Asian	10 (1.9%)	7 (1.9%)	1
Unknown	0 (0%)	0 (0%)	1
Tobacco mutational signature	3 (0.6%)	1 (0.3%)	0.647
*ICPI biomarkers*			
PD-L1 (DAKO 22C3)^c^	*n* = 141	*n* = 95	< 0.001
TPS < 1	31 (22%)	57 (60%)	–
TPS 1–49	59 (42%)	24 (25%)	–
TPS ≥50	51 (36%)	14 (15%)	–
TMB-H	27 (5.2%)	45 (12%)	<0.001
TMB median muts/Mb^a^	1.8 [0.9–3.8]	1.7 [0–5.0]	0.741
MSI-H	0 (0%)	29 (7.9%)	<0.001

*RET* rearranged during transfection, *NSCLC* non-small-cell lung cancer, *PD-L1* programmed death-ligand 1/Cluster of Differentiation 274, *TPS* Tumor Proportion Score, *TMB-H* tumor mutational burden-High, *MSI-H* microsatellite instability-high.

Other solid tumors exclude NSCLC.

See Supplementary Table S1 and S2 for further information.

^a^Wilcox rank-sum test.

^b^*p*-value adjusted for multiple comparisons.

^c^*χ*^2^ test.

The NSCLC *RET* fusion-positive cohort was significantly younger (median age = 64 vs 68; *P* < 0.001), had a higher female:male ratio (1.27 vs 1.02; P = 0.012) and had a higher frequency of specimens obtained from metastatic sites vs nonmetastatic sites (52% vs 43%; *P* = 0.002) when compared to the NSCLC *RET* fusion-negative cohort (Table 2). In the NSCLC cohort, patients with Central and South American, East Asian, and South Asian ancestry were more highly represented in the *RET* fusions-positive subset vs the *RET* fusion-negative subset (8.6% vs 5.1%, 11% vs 4.3%, 1.9% vs 0.6%, respectively, *P* < 0.001). Lastly, there was a significant decrease in the tobacco smoking mutational signature in the NSCLC *RET* fusion-positive cohort when compared to the NSCLC *RET* fusion-negative cohort (0.6% vs 13%; *P* < 0.001)^30^.

**Table 2:** Clinicopathologic and genomic biomarker characteristics comparing *RET* fusion-positive NSCLC and *RET* fusion-negative NSCLC.

	*RET* fus + NSCLC	*RET* fus- NSCLC	*p* value
	(*n* = 523)	(*n* = 61,310)	
Age median years [IQR]^a^	64 [55–71]	68 [60–75]	<0.001
Sex, female/male (%)	293/230 (56%/44%)	30,900/30,410 (50%/50%)	0.012
Specimen site			
Primary	249 (48%)	34,981 (57%)	<0.001
Metastatic	274 (52%)	26,203 (43%)	0.002
Unknown	0 (0%)	126 (0.2%)	0.304
Genetic ancestry^b^			
African	41 (7.8%)	5882 (9.6%)	0.200
Central and South American	45 (8.6%)	3148 (5.1%)	<0.001
East Asian	55 (11%)	2648 (4.3%)	<0.001
European	372 (71%)	49,232 (80.3%)	<0.001
South Asian	10 (1.9%)	389 (0.6%)	0.001
Unknown	0 (0%)	11 (<0.1%)	0.759
Tobacco mutational signature	3 (0.6%)	7776 (13%)	<0.001
*ICPI Biomarkers*			
PD-L1 (DAKO 22C3)^C^	*n* = 141	*n* = 23,640	<0.001
TPS < 1	31 (22%)	9026 (38%)	–
TPS 1–49	59 (42%)	7088 (30%)	–
TPS ≥50	51 (36%)	7526 (32%)	–
TMB-H	27 (5.2%)	21,896 (36%)	<0.001
TMB median muts/Mb^a^	1.8 [0.9–3.8]	7.0 [2.6–12.5]	<0.001
MSI-H	0 (0%)	219 (0.4%)	0.318

*RET* rearranged during transfection, *RET fus+* RET fusion-positive, *RET fus- RET* fusion-negative, *NSCLC* non-small-cell lung cancer, *PD-L1* Programmed death-ligand 1/Cluster of Differentiation 274, *TPS* Tumor Proportion Score, *TMB* tumor mutational burden, *MSI-H* microsatellite instability-high.

Other Solid Tumors exclude NSCLC.

^a^Wilcox rank-sum test.

^b^*p*-value adjusted for multiple comparisons.

^c^*χ*^2^ test.

For the other solid tumors *RET* fusion-positive cohort, we used NSCLC *RET* fusion positive cohort for inter-cohort comparison. Among 368 other solid tumors *RET* fusion-positive cases patients were significantly younger compared to NSCLC *RET* fusion-positive cases (median age = 61 vs 64; *P* < 0.001). In comparison to the NSCLC *RET* fusion-positive cohort, the other solid tumors *RET* fusion-positive cohort had a significantly higher prevalence of Central and South American and East Asian (8.6% vs 16%, 11% vs 3.8%, respectively, *P* < 0.01; Table 1). In addition, we examined the *RET* fusion positive to the *RET* fusion negative papillary thyroid carcinoma (PTC) cohorts and found that the age of the *RET* fusion positive PTC cohort was significantly younger then the *RET* fusion negative PTC cohort (33 vs 62 years old <0.001) (Supplementary Table 3). Lastly, we saw a trend in the different direction for colon adenocarcinoma, although a smaller absolute value difference (66 vs 60 years old, *p* < 0.001) (Supplementary Table 4).

#### RET in-frame fusion partners and breakpoints in NSCLC vs other solid tumors

All the *RET* fusion events in this cohort were in-frame events. Among all *RET* (10q11.21) fusion gene partners, 93% of genes reside in chromosome 10 across arms p and q. The top fusions partners identified in the NSCLC cohort were *KIF5B* (chr10 p11.22; 66%)*, CCDC6* (chr10 q21.2; 18.2%), *NCOA4* (chr10 q11.23; 2.9%), *TRIM24* (chr7 q34; 2%), *ERC1* (chr12 p13.33; 1%), and *KIAA1468* (chr18 q21.33; 1%) (Table 3 and Supplementary Fig. 1). On the other hand, more than half of the other solid tumors cohort was composed of *RET* fusions with gene fusion partners *NCOA4* (32.6%) and *CCDC6* (29.9%). Of note, the most common fusion in papillary thyroid carcinoma was *CCDC6-RET* and *NCOA4-RET* (41.3% and 35.8%, respectively). *KIF5B-RET* fusions were highly specific for NSCLC compared to other solid tumors (66% vs 6.3%; *P* < 0.001). In contrast *NCOA4-RET* (32.6% vs 2.9%, *P* < 0.001) and *CCDC6*-*RET* (30% vs 18.2%; *P* = 0.002) fusions was frequently seen among other solid tumors. In addition, 61 novel *RET* gene fusion partners were identified across both cohorts of patients (Table 3 and Supplementary Table 5).

**Table 3:** Comparative Genomics: Prevalence of *RET* fusions and partner genes among *RET* fusion-positive NSCLC and other solid tumors (fusions with at least 5 cases, for fusions with less than 5 cases please see Supplementary Table 3).

Fusions	NSCLC (*n* = 523) (%)	*n*	Other solid tumors (*n* = 368) (%)	*n*	Corrected *p*-value
*KIF5B-RET*^a^	66.0	346	6.3	23	<0.001
*CCDC6-RET*^a^	18.2	95	29.9	110	0.002
*NCOA4-RET*^a^	2.9	15	32.6	121	<0.001
*ERC1-RET*	1.0	5	2.7	10	0.705
*TRIM24-RET*	0.8	4	2.4	9	0.705
*TRIM33-RET*	0.6	3	1.1	4	0.767
*CSGALNACT2-RET* ^b^	0.0	0	1.6	6	0.135
*KIAA1217-RET*	0.2	1	1.4	5	0.705
*KIAA1468-RET*	0.8	5	0.5	2	1

^a^Significantly associated *RET* fusion gene partners among *RET* fusion-positive NSCLC and other solid tumors (excluding NSCLC).

^b^Novel *RET* fusion intergenic and intragenic gene partners.

**Fig. 2:**
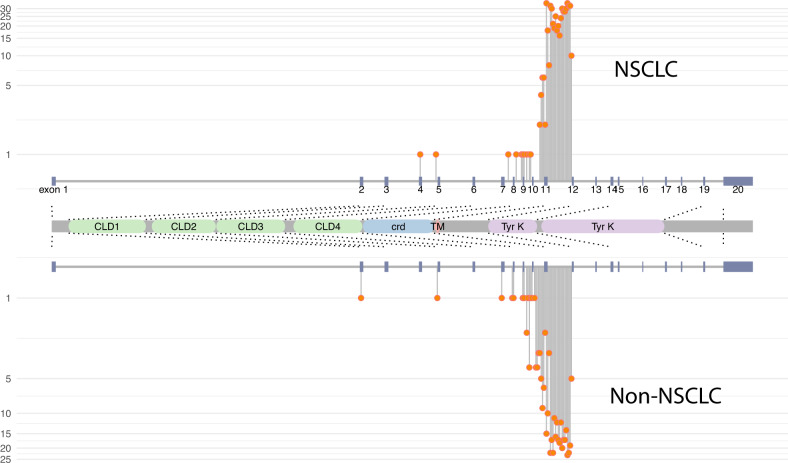
*RET* gene breakpoints were mainly clustered in the intron 11 and there is no difference in the distribution of *RET* breakpoint regions between NSCLC and other solid tumors *RET* fusion-positive cohorts. Representative lollipop plot scheme of *RET* gene [Chr10 (10q11.21)] demonstrating the frequency of *RET* gene fusion breakpoints among advanced *RET* fusion-positive NSCLC and other solid tumors (excluding NSCLC). Gray horizontal line indicates *RET* gene introns. Blue vertical bars indicate *RET* gene exons. Coding region extending from 43,077,259 to 43,128,266. Orange lollipops indicate prevalence of *RET* breakpoints binned by 100 bases for analytical reasons. Among 891 *RET* fusion events, 87% of break points occurred in intron 11 followed by intron 10 (5%) and exon 11 (4.8%). No significant differences were observed among *RET* break point regions between NSCLC and other solid tumors. *RET* extracellular region is coded by exons 1–10 and part of exon 11 (aa 29 to 635) responsible for CLD1, CLD2, CLD3, CLD4, and CRD (responsible for physiological receptor dimerization). A transmembrane region is coded by part of exon 11 (aa 636-657). Bipartite protein tyrosine kinase domains are coded by part of exon 12, exons 13-18 and part of exon 19 (aa 658 to 1114). Abbreviations: Rearranged during transfection (*RET*). Non-small-cell lung cancer (NSCLC). Cadherin-like domain (CLD). Cysteine-rich domain (CRD). Transmembrane domain (TM). Cytoplasmic intrinsic tyrosine kinase domain (Tyr K). Amino acids (aa).

We examined the primary break point regions in *RET* gene fusions. Among all the *RET* fusion-positive cohorts, the *RET* gene breakpoints were mainly clustered in the intron 11 (87%) followed by intron 10 (5%) and exon 11 (4.8%) (Fig. 2). There is no significant difference in the distribution of *RET* break point regions between NSCLC and other solid tumors. Similarly, in liquid biopsies, *RET* gene breakpoints were mainly clustered in intron 11 in both the NSCLC and other solid tumors *RET* fusion-positive cohorts (Supplementary Fig. 2).

#### Genes with genomic alterations in RET fusion defined cohorts

**Fig. 3:**
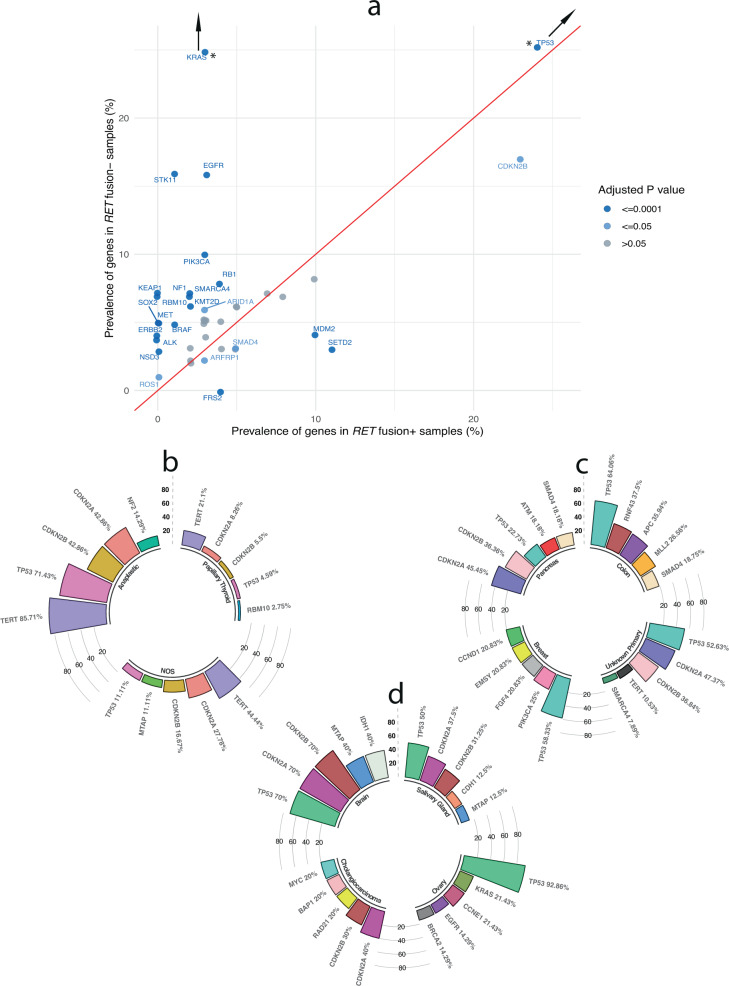
Prevalence of genes with genomic alterations differed amongst the *RET* fusion defined cohorts. **a** *RET* fusion driven NSCLC has a different genomic profile from *RET* wild-type NSCLC. Plot indicating prevalence of concurrent genomic variants among advanced *RET* fusion-positive NSCLC and *RET* fusion-negative NSCLC. Significant differences in the prevalence of genes with genomic alterations between *RET* fusion-positive NSCLC and *RET* fusion-negative NSCLC are indicated by a light blue (*p* ≤ 0.05) or dark blue dot (*p* ≤ 0.0001). *Off scale. Prevalence of most common 5 genes with genomic alterations among advanced *RET* fusion-positive, **b** thyroid cancer, **c** colon carcinoma, pancreatic carcinoma, breast carcinoma, unknown primary carcinoma, and **d** brain tumor, salivary gland carcinoma, ovarian carcinoma and cholangiocarcinoma.

We first interrogated frequently altered genes in the *RET* fusion-positive and *RET* fusion-negative NSCLC cohorts. The top 10 genes that are altered among *RET* fusion-positive NSCLC cases are *TP53* (43%)*, CDKN2A* (29%)*, CDKN2B* (23%)*, SETD2* (11%)*, MDM2* (10%)*, MYC* (10%)*, MTAP* (8%)*, NKX2-1* (7%), *NFKBIA* (5%), and *CDK4* (5%). In contrast, the top 10 genes that are altered among *RET* fusion-negative NSCLC patients are *TP53* (68%)*, KRAS* (31%)*, CDKN2A* (29%)*, CDKN2B* (17%)*, STK11* (16%)*, EGFR* (16%)*, MTAP* (13%)*, PIK3CA* (10%)*, RB1* (8%), and *MYC* (8%). Significantly more common co-occurring gene alterations among *RET* fusion-positive vs negative NSCLC patients include *CDKN2B, SETD2, MDM2, SMAD4, FRS2*, and *ARFRP1* (*P* < 0.05). Similarly, significantly common co-occurring gene alterations among *RET* fusion-negative vs positive NSCLC patients include *TP53, KRAS, STK11, EGFR, PIK3CA, RB1, NF1, SMARCA4, KEAP1, RBM10, ARID1A, KMT2D, SOX2, MET, BRAF, NSD3, ALK, ROS1, and ERBB2* (*P* < 0.001; Fig. 3a and Supplementary Table 6).

In *RET* fusion-positive other solid tumors*, TP53* (39%)*, CDKN2A* (22%)*, CDKN2B* (17%)*, TERT* (14%)*, APC* (8%)*, RNF43* (8%)*, PTEN* (7%), *MTAP* (6%)*, SMAD4* (6%), and *MLL2* (6%), are 10 most frequently altered genes (Supplementary Table 5). These genes varied amongst the various other solid tumors (Fig. 3b–d). When comparing the NSCLC *RET* fusion-positive with the other solid tumors *RET* fusion-positive cohort, we observed significant differences in several of the gene alteration frequencies (Supplementary Table 7). When comparing the *RET* fusion-positive PTC cohort, we observed a lower frequency of *BRAF, TERT, NRAS*, and *PIK3CA* genomic alterations when compared to the *RET* fusion negative PTC cohort (*P* < 0.05) (Supplementary Table 8). Lastly, when we examined the *RET* fusion-positive colon adenocarcinoma cohort, we observed a higher frequency of *RNF43, MLL2, CASP8, CREBBP, BCORL1, SPEN, SMARCA4, BRCA2, MSH3, PTCH1, QKI, EP300, LRP1B, CDH1*, and *FANCA*; but a lower frequency of *APC, KRAS, PIK3CA*, and *BRAF* genomic alterations when compared to the *RET* fusion-negative colon adenocarcinoma cohort (*P* < 0.05; Supplementary Table 9).

#### Co-NCCN guideline driver alterations among RET fusion positive NSCLC

We examined *RET* fusion positive NSCLC for targetable co-alterations listed in the National Comprehensive Cancer Network (NCCN) Guidelines. The specific NCCN genomic alterations that we examined were sensitizing *EGFR* mutations, *KRAS* G12C, *BRAF* V600E, *ERBB2* mutations, and *MET* exon 14 skipping mutations, *ALK* and *ROS1* rearrangements, *NTRK* fusions and *MET* amplifications. Overall, only 34 cases had co-occurring NCCN-NSCLC driver alterations. These included *EGFR* (3%, 17/223), *KRAS* (3%, 14/223), and *BRAF* (1%, 3/223).

#### Immune checkpoint inhibitor biomarkers

We examined immune checkpoint inhibitor (ICPI) biomarkers based on CGP and PD-L1 IHC. The NSCLC *RET* fusion-positive cohort had a significantly lower number of TMB-H cases and median TMB when compared to the NSCLC *RET* fusion-negative cohort (*P* < 0.001; Table 2). In comparison to the NSCLC *RET* fusion-positive cohort, the other solid tumors *RET* fusion-positive cohort had a significantly higher proportion of TMB-H cases, though the median TMB did not differ significantly (*P* < 0.001 & *P* = 0.741, respectively; Table 1). For the NSCLC *RET* fusion positive cases, 0% (0/27) TMB-H were MSI-H; and for the other solid tumor *RET* fusion positive cases, 53.3% (24/45) TMB-H were also MSI-H. This suggest that the differences in the higher TMB-H cases are likely due to the higher proportion of MSI-H in the other solid tumor *RET* fusion positive cases.

No cases had an MSI-H status in the overall NSCLC *RET* fusion-positive cohort. In comparison, 219 (0.4%) NSCLC *RET* fusion-negative cases were MSI-H and 29 (7.9%) other solid tumors *RET* fusion-positive cases were MSI-H (Tables 1–2**)**. Of note, 41.7% (25/60) of the *RET* fusion-positive colon adenocarcinomas had an MSI-H status which was significantly higher than the *RET* fusion negative colon adenocarcinoma (5.5%, 1805/32,938) (*p* < 0.001) (Supplementary Table 4). This same trend was seen in the prevalence of TMB-H status in colon adenocarcinoma (51.7% [31/60] vs 9.3% [3075/32,938], *p* < 0.001).

Among 141 *RET* fusion-positive NSCLC cases where we had also performed the PD-L1 22C3 CDx assay, 22% (31/141) had a negative TPS score (TPS < 1%), 42% (59/141) had a low positive TPS score (TPS 1–49) and 36% (51/141) had a high positive score (TPS ≥ 50). PD-L1 tumor cell expression in the NSCLC *RET* fusion-positive cohort was significantly higher than in the NSCLC *RET* fusion-negative cohort (*P* < 0.001) (Table 2). Of note, while DAKO 22C3 TPS is not a CDx in other solid tumors, we had 95 *RET* fusion-positive other solid tumor cases ran with the DAKO 22C3 and scored with TPS. In the other solid tumors cohort, 60% (57/95) had a negative TPS score (TPS < 1%), 25% (24/95) had a low positive TPS score (TPS 1–49) and 15% (14/95) had a high positive score (TPS ≥ 50; Table 1).

### Prevalence of tissue and liquid RET fusions detection

Among 891 total *RET* fusion positive cases, twenty-three cases were also tested with a liquid NGS assay. The median interval between specimen collection was 75 days, of which 10 out of 23 (43.5%) patients had a liquid assay performed after initial tissue-based NGS assay, 11 out of 23 (47.8%) had liquid assay as a primary comprehensive molecular NGS assay followed by tissue-based NGS and 2 of 23 (8.7%) had liquid and solid-based NGS at the same time point.

**Fig. 4:**
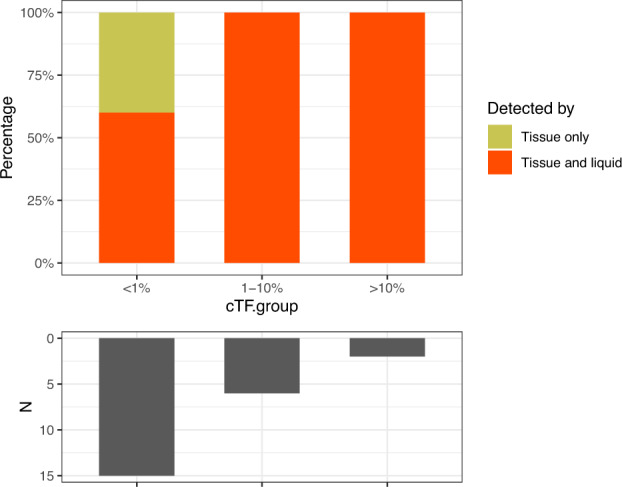
Amount of tumor shed is directly correlated with the ability of a liquid biopsy to detect *RET* fusions. The concordance of tissue and liquid testing stratified by composite tumor fraction (cTF) is as follows: 100% among 2 patients with greater than 10% cTF, 100% among 8 patients greater than 1% cTF and 40% (6) among 15 patients with less than 1% cTF.

Among 23 tissue *RET* fusion positive patients [20 (lung adenocarcinoma/NSCLC/lung cancer-NOS), 2 (carcinoma of unknown primary), 1 (prostatic adenocarcinoma)] with both tissue and liquid NGS, 14 (61%) patients had *RET* fusion detected on liquid assay. The concordance of tissue and liquid testing stratified by cTF is as follows: 100% among 2 patients with greater than 10% cTF, 100% among 8 patients greater than 1% cTF, and 40% (6) among 15 patients with less than 1% cTF (Fig. 4). Of the cases with cTF <1% and with detection of *RET* fusions with a liquid biopsy, the lowest cTF value was 0.27%. Lastly, among 9 patients with *RET* fusion positive tissue NGS but negative on liquid (all cTF <1%), 6 patients had no known somatic gene alterations detected in liquid and 3 patients had gene alterations (*TP53*, *BRCA1*, *JAK2* and *CHEK2*) with less than 0.5% variant allele frequency in liquid consistent with cTF <1%.

## Discussion

To the best of our knowledge, this study represents the largest single cohort of patients with *RET* fusion-positive solid tumors characterized by a DNA hybrid capture-based molecular assay. We observed that with a well-designed DNA based assay, the sensitivity of detecting *RET* fusions is comparable with the prevalence rates of The Cancer Genome Atlas (TCGA) network, which used a variety of molecular profiling techniques including: exome and whole genome DNA sequencing, RNA sequencing, miRNA sequencing, SNP arrays, DNA methylation arrays, and reverse phase protein arrays^31^. Specifically, the TCGA network yielded *RET* fusion positive PTC samples (*n* = 496) at 6.8%, whereas the *RET* fusion prevalence in our study was 9% (1208 sequenced PTC samples)^31^. Similarly, comprehensive molecular profiling of 229 lung adenocarcinoma by TCGA showed 2 samples (0.87%) with *RET* fusion in comparison to 1.14% *RET* fusion-positive among 39,922 lung adenocarcinomas in our study^26^. In addition, to the best of our knowledge, we have reported 61 novel *RET* fusions with intergenic or intragenic gene partners not yet reported in the literature^32,33^. These data point to the high sensitivity of detecting *RET* fusions by a well-designed DNA assay.

We defined the clinicopathologic and genomic landscape of this large cohort of tumors driven by *RET* fusions. Consistent with our findings, multiple studies have suggested that *RET* fusions in lung cancer correlate with adenocarcinoma histology, younger age, never smoker status and advanced disease^5,34,35^. A novel observation in the *RET* fusion-positive NSCLC patients was an enrichment of Central and South American, East Asian, and South Asian patients when compared to the *RET* fusion-negative NSCLC cohort. As expected, we observed that like other driver gene fusions (e.g. *ALK* and *ROS1)*, the majority of *RET* fusions are mutually exclusive with other primary driver alterations and the distribution of most common *RET* fusion partners [*KIF5B* 66%, *CCDC6* 18%, and others 16%] in NSCLC is similar to the existing *RET* registry studies on *RET* alterations^8^. Our findings also indicate no differences in the *RET* fusion breakpoints among NSCLC and other solid tumors. As previously described in *RET* fusion-positive CRC and similar to other fusions involving *ALK*, *ROS1*, and *NTRK*, we saw a high rate of MSI-H in patients with CRC driven by *RET* fusions^36,37^. Lastly, TMB is lower but the PD-L1 expression trended higher in the NSCLC *RET* fusion-positive cohort when compared to the NSCLC *RET* fusion negative cohort, which suggests for further efficacy evaluation of ICPI in *RET* fusion positive NSCLC.

Recently, liquid biopsy has emerged as an important tool for genomic profiling to guide clinical management of advanced NSCLC and other solid tumors. Studies describing somatic *RET* alterations detected using liquid NGS assays are rare^38^. In this context, the liquid assay utilized in this study detected 100% (8/8) of *RET* fusions among *RET* fusion-positive patients by tissue testing with cTF ≥1% and 40% (6/15) among *RET* fusion-positive patients by tissue testing with cTF <1%. While the number of patients with paired tissue and liquid testing was limited, this data suggests that when cTF is ≥1%, liquid biopsy can reliably detect *RET* fusions, and that when cTF is <1% *RET* fusion detection is still possible but negative results are less reliable. In this cohort, we detected a *RET* fusion in a case with a cTF value as low as 0.27%, suggesting that the assay was able to detect fusion even with very low amount of tumor shed.

This study has a few limitations. First, although this is the largest study to date to analyze co-occurring genomic alterations among *RET*-positive solid tumors, the cohort lacks full clinical annotation including therapeutic and systematic clinical follow-up information, stage of disease, smoking status, and reported race (though we infer the smoking status with the tobacco signature and race through the genetic ancestry of the patients). With additional clinical data, we could better characterize the efficacy of RET-inhibitors for various *RET* fusions, especially the novel fusions discovered. Furthermore, a small proportion of patients with *RET* fusion-positive NSCLC were also found to have other driver alterations, such as *EGFR* and *KRAS*. However, acquired *RET* fusions have been described as a mechanism of resistance to targeted therapies, such as *EGFR* inhibitors and without complete clinical annotation, it is difficult to determine if these were de novo alterations or acquired in the setting of targeted therapy. In addition, this *RET* fusion-positive study cohort is representative of primarily clinically advanced solid tumors and may not be representative of tumors in other clinical settings.

In conclusion, we present the clinicopathologic and genomic landscape of a large cohort of *RET* fusion positive tumors, including the discovery of 61 novel fusions, detected by a DNA tissue-based NGS assay. In addition, we observed that liquid biopsy-based NGS is highly sensitive for *RET* fusions at cTF ≥1%.

## Methods

### Patient cohort

A review of the Foundation Medicine research database was performed on patients that were tested with FoundationOne® or FoundationOne®CDx assays between August 2014 and December 2020 to review all patients whose tumor tissue harbored *RET* fusions. In addition, we queried the database to examine all patients tested with FoundationOne®LiquidCDx with *RET* fusions detected by tissue biopsy between August 2020-December 2021. This study was approved by the Western Institutional Review Board Protocol (No. 20152817) and the IRB granted a waiver of informed consent under 45 CFR § 46.116 based on review and determination that this research meets the following requirements: (i) the research involves no more than minimal risk to the subjects; (ii) the research could not practicably be carried out without the requested waiver; (iii) the waiver will not adversely affect the rights and welfare of the subjects. All patient cases in this study were sent to Foundation Medicine Inc. for comprehensive genomic profiling (CGP) during routine clinical care. Manual review of accompanying pathology reports was performed to extract demographic information of the patients and site of specimen.

### Tissue DNA sequencing assay

FoundationOne®CDx/FoundationOne® are tissue-based next generation sequencing (NGS) assays that uses a hybrid capture methodology and is performed in a Clinical Laboratory Improvement Amendments (CLIA)-certified and College of American Pathologists (CAP)-accredited laboratory (Foundation Medicine, Cambridge, MA and Morrisville, NC). FoundationOne®CDx/FoundationOne® detects base substitutions, insertion and deletion alterations (indels), and copy number alterations (CNAs) in up to 324 genes and select gene rearrangements as previously described^39^. Each sample is reviewed by a board-certified pathologist to assessed for % tumor nuclei/tumor volume adequacy and to assign a diagnosis to the sample. As previously described, the tumor mutational burden (TMB) was determined on up to 1.1 Mb of sequenced DNA and assessment of microsatellite instability (MSI)^40^ was performed from DNA sequencing on up to 114 loci^41,42^. TMB-high (H) was defined as ≥10 mutations/Megabase for the purposes of this study. As research use only, tobacco mutational signature was called as described by Zehir et al.^30^, and genetic ancestry was assessed to be of predominately African, European, Central and South American, South Asian, or East Asian genetic ancestry as previously described^43^.

### Liquid DNA sequencing assay

FoundationOne®LiquidCDx is a liquid biopsy CGP assay that utilizes a hybrid capture methodology and is performed in a CLIA-certified and CAP-accredited laboratory (Foundation Medicine, Cambridge, MA). Similar to FoundationOne®CDx/FoundationOne®, FoundationOne®LiquidCDx detects base substitutions, insertion, and deletion alterations (indels), and copy number alterations (CNAs) in up to 324 genes and select gene rearrangements^44^. An investigational composite tumor fraction (cTF), which merges two methods for estimation of tumor fraction (TF) was utilized as previously described^45–47^.

### RET fusion case selection

For this study, we included all *RET* fusions as detected by the FoundationOne®CDx/FoundationOne® and FoundationOne Liquid CDx assays (Foundation Medicine, Inc., Cambridge, MA). All rearrangements without a fusion partner were excluded from the analysis and only cases where the kinase domain of *RET* was preserved were included in this study.

### DAKO PD-L1 IHC 22C3 pharmDx assay

For a subset of samples (236 cases), *DAKO PD-L1 IHC 22C3 pharmDx Assay* was performed at Foundation Medicine concurrent to the FoundationOne®CDx/FoundationOne® assay. *DAKO PD-L1 IHC 22C3 pharmDx Assay* was run according to manufacturer instructions (Foundation Medicine, Inc, Morrisville, NC). All stained IHC slides were interpreted by board-certified pathologists utilizing DAKO’s tumor proportion score (TPS)^48^.

### Statistical analysis

We explored the clinical, pathologic, biomarker, and genomic differences between the different cohorts using Fisher’s exact test or *χ*^2^ test for categorical variables and Wilcox rank-sum test for continuous variables. *P*-value was adjusted for multiple comparisons using the Bonferroni method and *p* < 0.05 was considered significant^49^.

### Reporting summary

Further information on research design is available in the Nature Research Reporting Summary linked to this article.

## Supplementary information

Supplemental Figures and Tables

REPORTING SUMMARY

## Data Availability

The authors declare that all relevant aggregate data supporting the findings of this study are available within the article and its supplementary information files. In accordance with the Health Insurance Portability and Accountability Act, we do not have IRB approval or patient consent to share individualized patient genomic data, which contains potentially identifying or sensitive patient information and cannot be reported in a public data repository. Foundation Medicine is committed to collaborative data analysis and has well established and widely used mechanisms by which qualified researchers can query our core genomic database of >500,000 de-identified sequenced cancers. More information and mechanisms for data access can be obtained by contacting the corresponding author or the Foundation Medicine Data Governance Council at data.governance.council@foundationmedicine.com.
