# Supplementary material for: Genomic landscape of 891 RET fusions detected across diverse solid tumor types
Source: NPJ Precis Oncol. 2023 Jan 23;7:10. doi: 10.1038/s41698-023-00347-2 (PMC9870857; doi:10.1038/s41698-023-00347-2)
Supplement: Supplementary file 1 — Supplemental Figures and Tables [file 41698_2023_347_MOESM1_ESM.pdf]

Supplement

Supplemental Figure 1. The prevalence of fusion partners varied across tumor types. Pie chart of the prevalence of fusions partners in NSCLC, breast carcinoma, cancer of unknown primary, pancreatic adenocarcinoma, thyroid carcinoma, and colorectal adenocarcinoma.

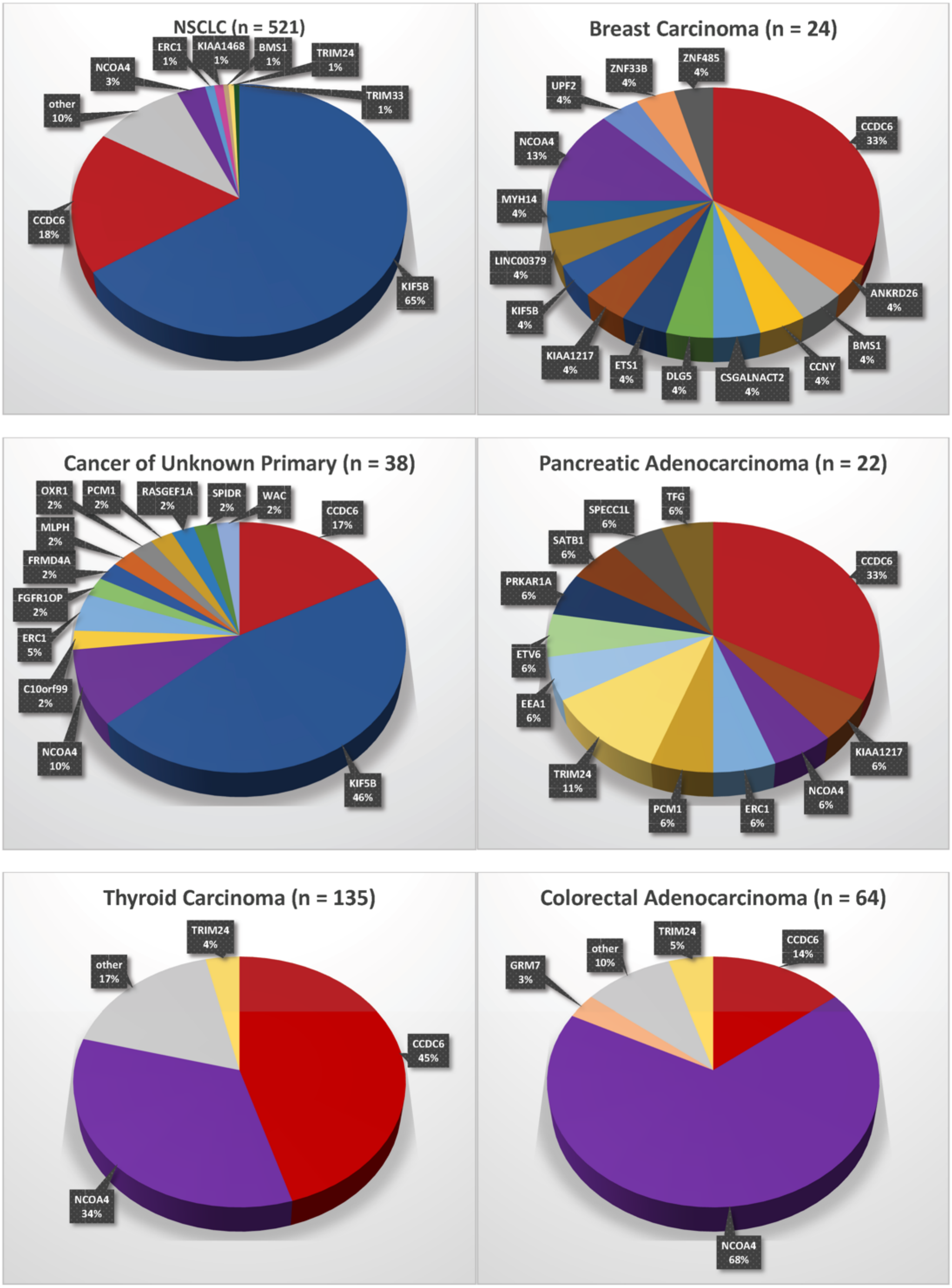

Supplemental Figure 2. In liquid biopsies, *RET* gene breakpoints were mainly clustered in intron 11 in both the NSCLC and other solid tumors *RET* fusion-positive cohorts. Representative lollipop plot scheme of *RET* gene [Chr10 (10q11.21)] demonstrating the frequency of *RET* gene fusion breakpoints among advanced *RET* fusion-positive NSCLC and other solid tumors (excluding NSCLC). Grey horizontal line indicates *RET* gene introns. Blue vertical bars indicate *RET* gene exons. Coding region extending from 43,077,259 to 43,128,266. Orange lollipops indicate prevalence of *RET* breakpoints binned by 100 bases for analytical reasons. *RET* extracellular region is coded by exons 1-10 and part of exon 11 (aa 29 to 635) responsible for CLD1, CLD2, CLD3, CLD4 and CRD (responsible for physiological receptor dimerization). A transmembrane region is coded by part of exon 11 (aa 636-657). Bipartite protein tyrosine kinase domains are coded by part of exon 12, exons 13-18 and part of exon 19 (aa 658 to 1114).

Abbreviations: Rearranged during transfection (*RET*). Non-small-cell lung cancer (NSCLC). Cadherin-like domain (CLD). Cysteine-rich domain (CRD). Transmembrane domain (TM). Cytoplasmic intrinsic tyrosine kinase domain (Tyr K). Amino acids (aa)

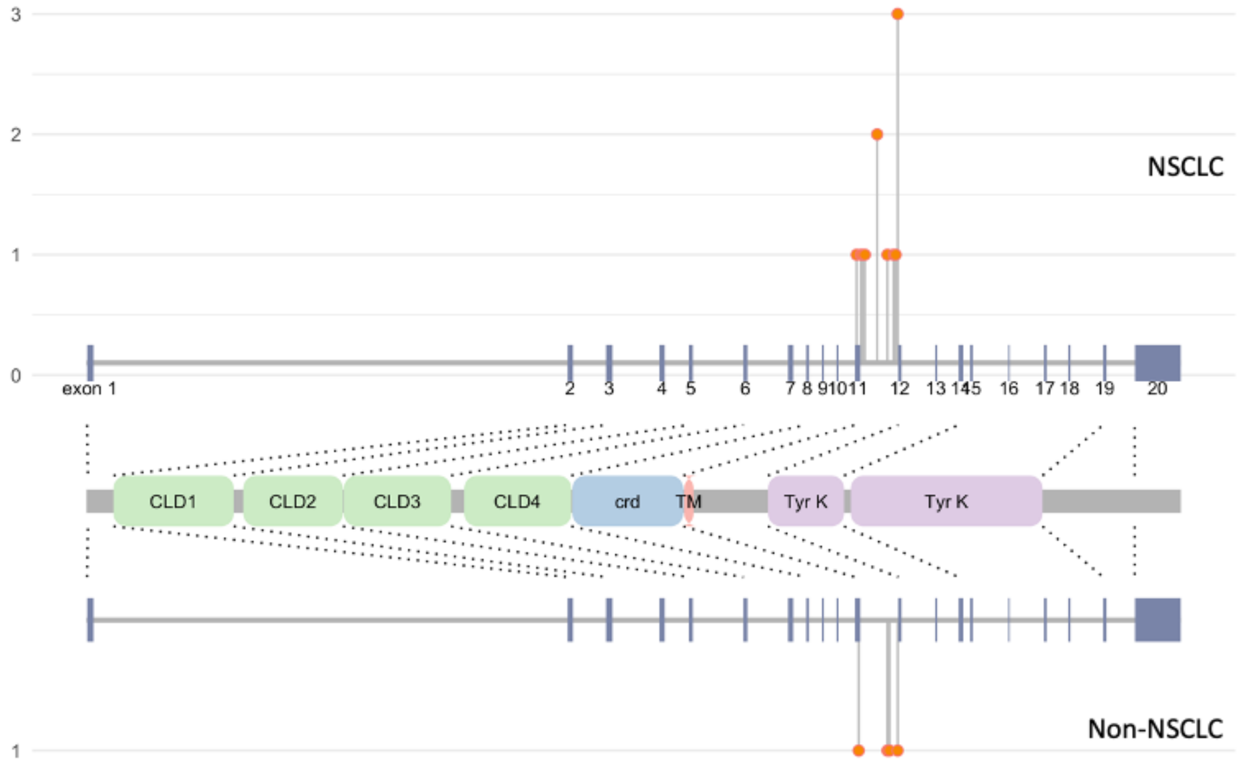

Supplemental Table 1. Frequency of *RET* fusion-positive advanced non-small-cell lung cancer (NSCLC) and other solid tumors (excluding NSCLC). Abbreviation: NOS, Not Otherwise Specified.

Diagnosis	Prevalence	*RET* fusion cases	Total cases
Lung adenocarcinoma	1.14%	455	39922
Thyroid papillary carcinoma	9.09%	109	1199
Colon adenocarcinoma	0.18%	60	32998
Lung non-small cell lung carcinoma (NOS)	0.54%	49	9117
Thyroid carcinoma (NOS)	3.02%	18	596
Pancreas ductal adenocarcinoma	0.11%	17	14940
Unknown primary adenocarcinoma	0.19%	16	8508
Unknown primary carcinoma (CUP)	0.31%	14	4520
Breast carcinoma (NOS)	0.06%	12	18978
Breast invasive ductal carcinoma	0.09%	11	11823
Lung squamous cell carcinoma	0.09%	9	11348
Intra-hepatic cholangiocarcinoma	0.15%	9	5993
Salivary gland carcinoma (NOS)	1.79%	8	446
Brain glioblastoma	0.12%	8	6671
Thyroid anaplastic carcinoma	1.33%	7	527
Ovary serous carcinoma	0.06%	5	10164
Ovary epithelial carcinoma	0.16%	5	3204
Prostate acinar adenocarcinoma	0.04%	5	5324
Esophagus adenocarcinoma	0.09%	4	12529
Salivary gland adenocarcinoma	1.24%	4	322
Bladder urothelial carcinoma	0.07%	4	5907
Salivary gland duct carcinoma	1.46%	3	205
Colon neuroendocrine carcinoma	0.72%	3	415
Lung sarcomatoid carcinoma	0.70%	3	431
Small intestine adenocarcinoma	0.37%	3	811
Lung large cell neuroendocrine carcinoma	0.31%	3	979
Ovary high grade serous carcinoma	0.14%	3	2190
Pancreatobiliary carcinoma	0.11%	3	2635
Lung small cell undifferentiated carcinoma	0.09%	3	3226
Stomach adenocarcinoma (NOS)	0.06%	3	4844
Lung atypical carcinoid	0.92%	2	217
Lung large cell carcinoma	0.87%	2	231
Lung adenosquamous carcinoma	0.43%	2	466
Pancreas islet cell tumor	0.22%	2	893
Pancreas carcinoma (NOS)	0.10%	2	1989

Unknown primary melanoma	0.05%	2	3849
Salivary gland mammary analogue secretory carcinoma	11.11%	1	9
Adrenal gland pheochromocytoma	1.37%	1	73
Brain Gliosarcoma	0.85%	1	118
Soft tissue leiomyosarcoma	0.78%	1	128
Pancreas acinar cell carcinoma	0.54%	1	185
Skin adnexal carcinoma	0.48%	1	210
Uterus endometrial adenocarcinoma mixed histology	0.44%	1	227
Esophagus carcinoma (NOS)	0.28%	1	360
Thyroid medullary carcinoma	0.27%	1	369
Unknown primary neuroendocrine tumor	0.19%	1	538
Ovary carcinosarcoma	0.17%	1	601
Unknown primary serous carcinoma	0.16%	1	631
Unknown primary (NOS)	0.13%	1	795
Unknown primary urothelial carcinoma	0.12%	1	842
Extra-hepatic cholangiocarcinoma	0.11%	1	873
Brain meningioma	0.11%	1	897
Skin squamous cell carcinoma	0.10%	1	971
Uterus carcinosarcoma	0.06%	1	1560
Unknown primary undifferentiated neuroendocrine carcinoma	0.06%	1	1611
Breast invasive lobular carcinoma	0.05%	1	1825
Unknown primary squamous cell carcinoma	0.05%	1	1839
Uterus endometrial adenocarcinoma (NOS)	0.02%	1	4019
Rectum adenocarcinoma	0.01%	1	6672
**Total**		**891**	**253770**

Supplemental Table 2. RET fusion-positive other solid tumors (excluding NSCLC) and respective diagnostic subtypes

**Tumor type**	**n**
**Thyroid carcinoma**	**135**
Thyroid anaplastic carcinoma	7
Thyroid carcinoma (NOS)	18
Thyroid medullary carcinoma	1
Thyroid papillary carcinoma	109
**Colorectal carcinoma**	**64**
Colon adenocarcinoma (CRC)	60
Colon neuroendocrine carcinoma	3
Rectum adenocarcinoma (CRC)	1
**Unknown primary cancer**	**38**
Unknown primary (NOS)	1
Unknown primary adenocarcinoma	16
Unknown primary carcinoma (cup) (NOS)	14
Unknown primary melanoma	2
Unknown primary neuroendocrine tumor	1
Unknown primary serous carcinoma	1
Unknown primary squamous cell carcinoma (SCC)	1
Unknown primary undifferentiated neuroendocrine carcinoma	1
Unknown primary urothelial carcinoma	1
**Breast carcinoma**	**24**
Breast carcinoma (NOS)	12
Breast invasive ductal carcinoma (IDC)	11
Breast invasive lobular carcinoma (ILC)	1
**Pancreatic cancer**	**22**
Pancreas acinar cell carcinoma	1
Pancreas carcinoma (NOS)	2
Pancreas ductal adenocarcinoma	17
Pancreas islet cell tumor	2
**Salivary gland carcinoma**	**16**
Salivary gland adenocarcinoma	4
Salivary gland carcinoma (NOS)	8
Salivary gland duct carcinoma	3
Salivary gland mammary analogue secretory carcinoma (masc)	
**Ovarian carcinoma**	**14**
Ovary carcinosarcoma	1
Ovary epithelial carcinoma	5

Ovary high grade serous carcinoma	3
Ovary serous carcinoma	5
**Cholangiocarcinoma**	**10**
Extra-hepatic cholangiocarcinoma	1
Intra-hepatic cholangiocarcinoma	9
**Brain cancer**	**10**
Brain glioblastoma (GBM)	8
Brain gliosarcoma	1
Brain meningioma	1
**Lung cancer (excluding NSCLC)**	**5**
Lung atypical carcinoid	2
Lung small cell undifferentiated carcinoma	3
**Prostate acinar adenocarcinoma**	**5**
**Esophageal carcinoma**	**5**
Esophagus adenocarcinoma	4
Esophagus carcinoma (NOS)	1
**Bladder urothelial (transitional cell) carcinoma**	**4**
**Uterine carcinoma**	**3**
Uterus carcinosarcoma	1
Uterus endometrial adenocarcinoma (NOS)	1
Uterus endometrial adenocarcinoma mixed histology	1
**Stomach adenocarcinoma (NOS)**	**3**
**Small intestine adenocarcinoma**	**3**
**Pancreatobiliary carcinoma**	**3**
**Skin carcinoma**	**2**
Skin adnexal carcinoma	1
Skin squamous cell carcinoma (SCC)	1
**Adrenal gland pheochromocytoma**	**1**
**Soft tissue leiomyosarcoma**	**1**
**Total**	**368**

Supplemental Table 3. Comparison of clinicopathologic and genomic biomarker characteristics of *RET* fusion-positive and *RET* fusion-negative Papillary Thyroid Carcinoma

	RET fus+ Papillary Thyroid Carcinoma (n=109)	RET fus- Papillary Thyroid Carcinoma (n=1090)	p value
**Age, median years [IQR]***	33 [22-56]	62 [52-71]	<0.001
**Sex, Female/Male**	68/41 (62%/38%)	560/530 (51%/49%)	0.028
**Specimen Site**			0.433
Primary	49 (45%)	422 (39%)	
Metastatic	52 (48%)	571 (52%)	
Unknown	8 (7%)	97 (9%)	
**Genetic Ancestry****			0.159
African	10 (9%)	55 (5%)	
Central and South American	24 (22%)	202 (19%)	
East Asian	5 (5%)	79 (7%)	
European	67 (62%)	739 (68%)	
South Asian	3 (3%)	15 (1%)	
ICPI biomarkers			
**PD-L1 (DAKO 22C3)*****	n=27	n=261	0.992
<1	14 (52%)	135 (52%)	
1-49	9 (33%)	85 (33%)	
≥50	4 (15%)	41 (16%)	
**TMB-H**	0 (0.0%)	17 (1.6%)	0.189
**TMB, median muts/Mb**	0 [0.0-1.3]	1.3 [0.0-2.5]	0.7413
**MSI-H**	0 (0%)	0 (0%)	1

Abbreviations: Rearranged during transfection (*RET*). Non-small-cell lung cancer (NSCLC). Programmed death-ligand 1/Cluster of Differentiation 274 (PD-L1). Tumor Proportion Score (TPS). Tumor mutational burden-High (TMB-H). Microsatellite instability-high (MSI-H). Other Solid Tumors exclude NSCLC.

*Wilcox Rank Sum Test

**p-value adjusted for multiple comparisons;

***Chi-squared Test

Supplemental Table 4. Comparison of clinicopathologic and genomic biomarker characteristics of *RET* fusion-positive and *RET* fusion-negative colon adenocarcinoma

	RET fus+ Colon adenocarcinoma (n=60)	RET fus- Colon adenocarcinoma (n=32,938)	p value
**Age, median years [IQR]***	66 [63-75]	60 [51-69]	< 0.001
**Sex, Female/Male/unknown**	30/30/0 (50%/50%/0)	15,343/17,573/22 (47%/53%/0.1%)	0.854
**Specimen Site**			0.021
Primary	40 (66.7%)	16090 (48.8%)	
Metastatic	20 (33.3%)	16753 (50.9%)	
Unknown	0 (0.0%)	95 (0.3%)	
**Genetic ancestry****			0.812
African	5 (8.3%)	4067 (12.3%)	
Central and South American	8 (13.3%)	3182 (9.7%)	
East Asian	3 (5.0%)	1376 (4.2%)	
European	43 (71.7%)	24033 (73.0%)	
South Asian	1 (1.7%)	271 (0.8%)	
ICPI biomarkers			
**PD-L1 (Dako 22C3)*****	n=19	n=7234	0.006
<1	12 (63%)	6216 (86%)	
1-49	7 (37%)	907 (13%)	
≥50	0 (0%)	111 (2%)	
**TMB-H**	31 (51.7%)	3075 (9.3%)	< 0.001
**TMB, median muts/Mb**	10 [5.2-40]	3.8 [2.5-6.1]	< 0.001
**MSI-H**	25 (41.7%)	1805 (5.5%)	< 0.001

Abbreviations: Rearranged during transfection (*RET*). Non-small-cell lung cancer (NSCLC). Programmed death-ligand 1/Cluster of Differentiation 274 (PD-L1). Tumor Proportion Score (TPS). Tumor mutational burden-High (TMB-H). Microsatellite instability-high (MSI-H). Other Solid Tumors exclude NSCLC.

*Wilcox Rank Sum Test

**p-value adjusted for multiple comparisons;

***Chi-squared Test

Supplemental Table 5. Comparative Genomics: Prevalence of *RET* fusions and partner genes among *RET* fusion-positive NSCLC and other solid tumors (fusions with <5 cases, for fusions with more than 5 cases please see Table 3). *Significantly associated *RET* fusion gene partners among *RET* fusion-positive NSCLC and other solid tumors (excluding NSCLC). ⊕Novel *RET* fusion intergenic and intragenic gene partners.

Fusions	NSCLC (n=523)	n	Other solid tumors (n=368)	n	Corrected p-value
EML4-RET	0.0%	0	1.1%	4	0.533
GOLGA5-RET	0.0%	0	1.1%	4	0.533
BMS1-RET ⊕	0.6%	3	0.3%	1	0.983
PCM1-RET	0.0%	0	0.8%	3	0.705
SNRNP70-RET	0.2%	1	0.5%	2	0.883
RASGEF1A-RET ⊕	0.4%	2	0.3%	1	1
ABI3BP-RET	0.0%	0	0.5%	2	0.705
DLG5-RET	0.0%	0	0.5%	2	0.705
ETV6-RET	0.0%	0	0.5%	2	0.705
TAF3-RET ⊕	0.0%	0	0.5%	2	0.705
ZNF33B-RET	0.0%	0	0.5%	2	0.705
WAC-RET	0.2%	1	0.3%	1	1
EEA1-RET	0.2%	1	0.3%	1	1
RUFY2-RET	0.2%	1	0.3%	1	1
TNIP1-RET	0.2%	1	0.3%	1	1
KIF13A-RET	0.4%	2	0.0%	0	0.805
LSM14A-RET	0.4%	2	0.0%	0	0.805
PARD3-RET	0.4%	2	0.0%	0	0.805
PCDH15-RET ⊕	0.4%	2	0.0%	0	0.805
ARHGAP19-RET ⊕	0.2%	1	0.0%	0	1
ACPP-RET	0.0%	0	0.3%	1	0.705
ADAMTS14-RET ⊕	0.0%	0	0.3%	1	0.705
ADK-RET ⊕	0.0%	0	0.3%	1	0.705
AGBL4-RET	0.0%	0	0.3%	1	0.705
ALOX5-RET ⊕	0.0%	0	0.3%	1	0.705
ANKRD26-RET	0.0%	0	0.3%	1	0.705
C10orf99-RET ⊕	0.0%	0	0.3%	1	0.705

CASC8-RET ⊕	0.0%	0	0.3%	1	0.705
CCDC186-RET ⊕	0.0%	0	0.3%	1	0.705
CCNY-RET ⊕	0.0%	0	0.3%	1	0.705
CEP135-RET ⊕	0.0%	0	0.3%	1	0.705
EBF1-RET ⊕	0.0%	0	0.3%	1	0.705
ETS1-RET	0.0%	0	0.3%	1	0.705
FGFR1OP-RET ⊕	0.0%	0	0.3%	1	0.705
FRMD4A-RET	0.0%	0	0.3%	1	0.705
GNA14-RET	0.0%	0	0.3%	1	0.705
GRM7-RET ⊕	0.0%	0	0.3%	1	0.705
HOOK1-RET ⊕	0.0%	0	0.3%	1	0.705
IFT74-RET ⊕	0.0%	0	0.3%	1	0.705
LINC00379-RET ⊕	0.0%	0	0.3%	1	0.705
LMNA-RET ⊕	0.0%	0	0.3%	1	0.705
LRMDA-RET ⊕	0.0%	0	0.3%	1	0.705
MGEA5-RET ⊕	0.0%	0	0.3%	1	0.705
MLPH-RET ⊕	0.0%	0	0.3%	1	0.705
MYH14-RET ⊕	0.0%	0	0.3%	1	0.705
NTRK2-RET ⊕	0.0%	0	0.3%	1	0.705
OLFM4-RET	0.0%	0	0.3%	1	0.705
OXR1-RET ⊕	0.0%	0	0.3%	1	0.705
PDE4D-RET ⊕	0.0%	0	0.3%	1	0.705
PRKAR1A-RET	0.0%	0	0.3%	1	0.705
PRKG1-RET	0.0%	0	0.3%	1	0.705
RASAL2-RET	0.0%	0	0.3%	1	0.705
RBMS3-RET ⊕	0.0%	0	0.3%	1	0.705
REEP3-RET ⊕	0.0%	0	0.3%	1	0.705
RRBP1-RET	0.0%	0	0.3%	1	0.705
SAMD4A-RET ⊕	0.0%	0	0.3%	1	0.705
SATB1-RET	0.0%	0	0.3%	1	0.705
SLC36A2-RET ⊕	0.0%	0	0.3%	1	0.705
SORBS1-RET	0.0%	0	0.3%	1	0.705
SPECC1L-RET	0.0%	0	0.3%	1	0.705
SPIDR-RET ⊕	0.0%	0	0.3%	1	0.705

SQSTM1-RET	0.0%	0	0.3%	1	0.705
TFG-RET	0.0%	0	0.3%	1	0.705
TNIP2-RET	0.0%	0	0.3%	1	0.705
TRIM27-RET	0.0%	0	0.3%	1	0.705
UPF2-RET ⊕	0.0%	0	0.3%	1	0.705
VSTM4-RET ⊕	0.0%	0	0.3%	1	0.705
ZNF485-RET ⊕	0.0%	0	0.3%	1	0.705
ZNF487-RET ⊕	0.0%	0	0.3%	1	0.705
ATRNL1-RET ⊕	0.2%	1	0.0%	0	1
CCDC88C-RET	0.2%	1	0.0%	0	1
CHAT-RET ⊕	0.2%	1	0.0%	0	1
CLIP1-RET	0.2%	1	0.0%	0	1
CNTN1-RET	0.2%	1	0.0%	0	1
CPEB3-RET ⊕	0.2%	2	0.0%	0	0.805
DCC-RET ⊕	0.2%	1	0.0%	0	1
DOCK1-RET	0.2%	1	0.0%	0	1
DSP-RET ⊕	0.2%	1	0.0%	0	1
ELMO1-RET	0.2%	1	0.0%	0	1
GAS2-RET ⊕	0.2%	1	0.0%	0	1
LCOR-RET ⊕	0.2%	1	0.0%	0	1
MGMT-RET ⊕	0.2%	1	0.0%	0	1
MKX-RET ⊕	0.2%	1	0.0%	0	1
MYH9-RET ⊕	0.2%	1	0.0%	0	1
NPAS3-RET ⊕	0.2%	1	0.0%	0	1
OPTN-RET ⊕	0.2%	1	0.0%	0	1
PHYHIPL-RET ⊕	0.2%	1	0.0%	0	1
PIBF1-RET ⊕	0.2%	1	0.0%	0	1
RBPMS-RET	0.2%	1	0.0%	0	1
SGIP1-RET ⊕	0.2%	1	0.0%	0	1
SH2D3A-RET ⊕	0.2%	1	0.0%	0	1
SLC12A2-RET ⊕	0.2%	1	0.0%	0	1
TPR-RET	0.2%	1	0.0%	0	1
TRIM67-RET ⊕	0.2%	1	0.0%	0	1
UBE2D1-RET	0.2%	1	0.0%	0	1

WWTR1-RET ⊕	0.2%	1	0.0%	0	1
ZNF239-RET ⊕	0.2%	1	0.0%	0	1
ZSWIM6-RET ⊕	0.2%	1	0.0%	0	1
RAI14-RET ⊕	0.2%	1	0.0%	0	1
RAD1-RET ⊕	0.2%	1	0.0%	0	1
CCBE1-RET	0.2%	1	0.0%	0	1
NAALADL2-RET ⊕	0.2%	0	0.0%	1	0.705

Supplemental Table 6. Comparative Genomics: Co-occurring genomic alterations and significant associations among *RET* fusion-positive NSCLC and *RET* fusion-negative NSCLC *Includes any genes in the top 30 most frequently mutated in either group. **Targetable driver fusions/amplifications in NSCLC

Gene*	*RET* fus+ NSCLC (n=523)	%	*RET* fus- NSCLC (n=61,310)	%	Corrected P-Value
TP53	223	43%	41446	68%	<0.001
KRAS	14	3%	18945	31%	<0.001
CDKN2A	153	29%	17903	29%	1
CDKN2B	120	23%	10545	17%	0.002
STK11	5	1%	9687	16%	<0.001
EGFR	17	3%	9626	16%	<0.001
MTAP	40	8%	4389	7%	0.739
PIK3CA	14	3%	6315	10%	<0.001
RB1	19	4%	5027	8%	<0.001
MYC	52	10%	4843	8%	0.118
NF1	11	2%	4476	7%	<0.001
NKX2-1	37	7%	4476	7%	0.955
SMARCA4	9	2%	4353	7%	<0.001
KEAP1	2	0%	4292	7%	<0.001
RBM10	2	0%	4292	7%	<0.001
ARID1A	16	3%	3924	6%	0.002
NFKBIA	28	5%	3679	6%	0.728
PTEN	25	5%	3556	6%	0.473
KMT2D	8	2%	3495	6%	<0.001
SOX2	1	0%	3311	5%	<0.001
MET	2	0%	3249	5%	<0.001
BRAF	3	1%	3249	5%	<0.001
RAD21	13	2%	1716	3%	0.828
RICTOR	17	3%	3127	5%	0.087
CCND1	17	3%	3004	5%	0.116
DNMT3A	16	3%	3004	5%	0.083
NSD3	1	0%	1584	3%	<0.001
FGF19	16	3%	2820	5%	0.144
ATM	21	4%	2820	5%	0.697
FGF3	15	3%	2820	5%	0.087
SETD2	60	11%	1778	3%	<0.001
MDM2	53	10%	2698	4%	<0.001
CDK4	27	5%	2085	3%	0.063
SMAD4	26	5%	1839	3%	0.026
CTNNB1	23	4%	1839	3%	0.101

FRS2	22	4%	0	0%	<0.001
ARFRP1	15	3%	920	2%	0.030
FGF4	15	3%	2698	4%	0.138
GNAS	13	2%	1410	2%	0.826
CHEK2	13	2%	1042	2%	0.210
ALK **	0	0%	1839	3%	<0.001
ROS1 **	0	0%	552	1%	0.030
ERBB2 **	0	0%	2452	4%	4.43E-09

Supplemental Table 7. Comparative Genomics: Genomic alterations and significant associations among *RET* fusion-positive NSCLC and other solid tumors cohort

Gene	NSCLC (n=523)	n	Other Solid Tumors (n=368)	n	Corrected P-Value
TP53	42.6%	223	39.1%	144	1
CDKN2A	29.3%	153	22.0%	81	0.497
CDKN2B	22.9%	120	16.8%	62	0.847
MYC	9.9%	52	5.4%	20	0.519
SETD2	11.5%	60	2.2%	8	<0.001
MTAP	7.6%	40	6.3%	23	1
TERT	1.9%	10	13.6%	50	<0.001
MDM2	10.1%	53	0.8%	3	<0.001
PTEN	4.8%	25	6.5%	24	1
SMAD4	5.0%	26	6.0%	22	1
NKX2-1	7.1%	37	1.1%	4	<0.001
APC	1.7%	9	7.9%	29	<0.001
ARID1A	3.1%	16	6.0%	22	1
ATM	4.0%	21	3.8%	14	1
CCND1	3.3%	17	4.3%	16	1
RNF43	0.8%	4	7.6%	28	<0.001
FGF19	3.1%	16	4.1%	15	1
NFKBIA	5.4%	28	0.8%	3	0.004
MLL2	1.5%	8	6.0%	22	0.010
FGF3	2.9%	15	4.1%	15	1
FGF4	2.9%	15	4.1%	15	1
PIK3CA	2.7%	14	4.1%	15	1
CDK4	5.2%	27	0.3%	1	<0.001
RB1	3.6%	19	2.2%	8	1
CTNNB1	4.4%	23	1.1%	4	0.139
EGFR	3.3%	17	2.4%	9	1
FRS2	4.2%	22	1.1%	4	0.223
DNMT3A	3.1%	16	1.9%	7	1
GNAS	2.5%	13	2.4%	9	1
RICTOR	3.3%	17	1.4%	5	1

Supplemental Table 8. Comparative Genomics: Genomic alterations and significant associations among *RET* fusion-positive and fusion-negative papillary thyroid carcinoma (PTC) *Includes any genes in the top 30 most frequently mutated in either group.

**Gene***	*RET* fusion positive PTC (n=109)	**n**	**RET fusion negative PTC (n=1090)**	**n**	**corrected p-value**
BRAF	0%	0	78%	855	<0.001
TERT	21%	23	64%	702	<0.001
CDKN2A	8%	9	10%	107	0.829
TP53	5%	5	10%	106	0.417
NRAS	0%	0	7%	81	0.006
PIK3CA	0%	0	7%	75	0.011
CDKN2B	6%	6	6%	61	1
RBM10	3%	3	5%	50	0.816
DNMT3A	1%	1	4%	49	0.417
ATM	1%	1	3%	37	0.619
STAG2	0%	0	2%	26	0.564
CHEK2	3%	3	2%	23	0.829
MTAP	3%	3	2%	22	0.816
NTRK1	0%	0	2%	20	0.619
KRAS	0%	0	2%	19	0.749
ARID1A	0%	0	2%	18	0.749
BCOR	0%	0	2%	18	0.749
TET2	1%	1	2%	17	1
MEN1	0%	0	2%	17	0.749
PTEN	2%	2	2%	17	0.829
MUTYH	2%	2	1%	16	0.829
HRAS	1%	1	1%	15	1
NUP93	0%	0	1%	14	0.816
AKT1	0%	0	1%	13	0.816
U2AF1	0%	0	1%	12	0.816
AKT2	0%	0	1%	12	0.816
ASXL1	0%	0	1%	11	0.816
BCORL1	1%	1	1%	10	1
NF2	3%	3	1%	10	0.467
APC	0%	0	1%	10	0.816
TBX3	2%	2	0%	2	0.305

BRCA1	2%	2	1%	10	0.698
EP300	2%	2	1%	7	0.617
SETD2	2%	2	1%	6	0.564
BRCA2	2%	2	1%	8	0.619

Supplemental Table 9. Comparative Genomics: Genomic alterations and significant associations among *RET* fusion-positive and fusion-negative colon adenocarcinoma *Includes any genes in the top 30 most frequently mutated in either group.

**Gene***	***RET* fusion positive colon adenocarcinoma (n=60)**	**n**	***RET* fusion negative colon adenocarcinoma (n=32,938)**	**n**	**corrected p-value**
APC	37%	22	77%	25288	<0.001
TP53	65%	39	75%	24644	0.188
KRAS	2%	1	50%	16523	<0.001
PIK3CA	3%	2	19%	6368	0.002
SMAD4	20%	12	16%	5248	0.467
SOX9	15%	9	11%	3647	0.387
BRAF	0%	0	10%	3416	0.007
FBXW7	13%	8	9%	3106	0.354
PTEN	10%	6	9%	2804	0.670
MYC	7%	4	8%	2501	1.000
FLT3	2%	1	7%	2385	0.216
FAM123B	8%	5	7%	2377	0.663
ARID1A	17%	10	7%	2359	0.023
CDK8	2%	1	7%	2345	0.216
GNAS	3%	2	7%	2176	0.513
RNF43	40%	24	6%	2036	<0.001
ATM	7%	4	5%	1731	0.609
BCL2L1	0%	0	5%	1599	0.216
ERBB2	3%	2	5%	1556	1.000
ASXL1	8%	5	4%	1468	0.303
CTNNB1	2%	1	4%	1468	0.587
NRAS	0%	0	4%	1345	0.291
PIK3R1	5%	3	4%	1316	0.587
MLL2	28%	17	4%	1314	<0.001
IRS2	0%	0	3%	1113	0.354
SMAD2	0%	0	3%	1066	0.354
ZNF217	0%	0	3%	1039	0.354
AURKA	0%	0	3%	1030	0.354
ARFRP1	0%	0	3%	991	0.354
SRC	0%	0	3%	946	0.508
CASP8	17%	10	2%	511	<0.001
CREBBP	13%	8	2%	810	0.001

BCORL1	12%	7	3%	881	0.004
SPEN	12%	7	1%	359	<0.001
SMARCA4	12%	7	2%	517	<0.001
BRCA2	10%	6	3%	882	0.014
MSH3	10%	6	2%	638	0.004
PTCH1	10%	6	2%	516	0.002
QKI	10%	6	1%	389	<0.001
EP300	8%	5	2%	589	0.012
LRP1B	8%	5	2%	563	0.011
CDKN2A	7%	4	3%	897	0.159
NF1	7%	4	2%	817	0.127
CDH1	7%	4	1%	406	0.016
FLCN	7%	4	2%	625	0.062
MSH6	7%	4	2%	693	0.081
FANCA	7%	4	1%	267	0.005
